# Effects of low occlusal loading on the neuromuscular behavioral development of cortically-elicited jaw movements in growing rats

**DOI:** 10.1038/s41598-021-86581-9

**Published:** 2021-03-30

**Authors:** Phyo Thura Aung, Chiho Kato, Akiyo Fujita, Yasunori Abe, Takuya Ogawa, Hideyuki Ishidori, Hidemasa Okihara, Satoshi Kokai, Takashi Ono

**Affiliations:** Department of Orthodontic Science, Graduate School, Tokyo Medical and Dental University (TMDU), 1-5-45 Yushima Bunkyo-ku, Tokyo, 113-8549 Japan

**Keywords:** Neurophysiology, Cognitive neuroscience, Feeding behaviour

## Abstract

The effect of altered occlusal force on masticatory-related neuromuscular control, which projects from the anterior part of the cortical masticatory area (A-CMA), during growth remains unclear. This study sought to evaluate the effect of occlusal hypofunction on neuromuscular development of jaw muscle activities and cortically-induced rhythmic jaw movements (RJMs) in growing rats. Sixty-four 2-week-old male albino Wistar rats were divided into the control (fed normal diet) and experimental (fed soft diet) groups soon after weaning. Electromyographic activity was recorded at 5, 7, 9, and 11 weeks from the right masseter and anterior digastric along with RJMs. We found a significantly longer onset latency and smaller peak-to-peak amplitude in the experimental group than that in the control group. The RJMs showed an increase in gape size and lateral excursion until up to 9 weeks in both groups. However, both the average gape size and lateral excursion were significantly smaller in the experimental group than that in the control group after 9 weeks. The jaw movement pattern also showed a significant decrease at the maximum opening period in the experimental group. Our findings indicate that inadequate occlusal function during growth alters neuromuscular control of masticatory behaviors and impairs the pattern of RJMs.

## Introduction

Locomotor activities, including walking, breathing, chewing, and swallowing, are guided under the full/partial control of brainstem neural circuits known as central pattern generators (CPGs) influenced by cortical motor outputs from higher brain areas. Motor outputs from cortical areas influence rhythmic motor behaviors in various parts of the body, including the orofacial region^1^. These behaviors are important for motor activity and significantly contribute to sensory inputs as well as the structural and functional development of the brain. The interplay between central motor areas and sensory influences is essential for the production of adaptive behaviors for motor programs.

Sensory feedback plays an important role in the neural control of motor functions^2^. The activities of sensory neurons are mainly related to force control in movement tasks. For example, the unloading action of limb muscles inhibits the activity of motoneurons during movement tasks^3^. Furthermore, a study on rats has suggested that unloading activity of the hindlimb strongly modifies both motor behavior and skilled motor activity, producing alterations in locomotor activity^4^. Taken together, these results suggest that cortical mechanisms including sensorimotor integration play a critical role in the generation and control of locomotor behavior.

According to the findings of a brain mapping study, a relatively large portion of the cortical motor area regulates the voluntary movements of orofacial behaviors^5^. In fact, bilateral peripheral inputs from orofacial tissues modulate the neural organization of the cortical motor area and regulate orofacial behaviors to produce appropriate motor responses. However, motor representation decreases following peripheral nerve injury and neuronal activity increases in relation to movement training^6^. Furthermore, it has been shown that changes in orofacial environments, such as altered dental occlusion, increased vertical occlusal dimension, tooth extraction, lingual nerve injury, and nasal obstruction, can induce neuroplastic changes in jaw and tongue motor representation within the motor cortex^7–11^.

Moreover, altered masticatory function due to the long-term consumption of soft diet affects craniofacial morphology^12,13^ and differentiation in the development of muscle fibers in animals^14^. Importantly, a recent study has reported that a soft diet can lead to a variety of unfavorable health outcomes including changes in systemic, mental, and physical behaviors of the body^15^. However, it remains controversial whether occlusal hypofunction in neuromuscular control of the jaw muscles is specifically induced in association with changes in the cortical motor area, and whether these changes are temporary or permanent under conditions of altered occlusal behavior.

Previous electrophysiological studies have demonstrated that the relatively large parts of the cortical motor areas, the so-called cortical masticatory area (CMA), generate orofacial behaviors^5,16,17^. Specifically, electromyographic (EMG) activities of the masticatory muscles and cortically-induced rhythmic jaw movements (RJMs) generated by the CMA have been used as an experimental model for investigating the neural systems involved in masticatory behavior^16,18^. In the CMA, the anterior and posterior parts generate different masticatory behaviors; these areas are referred to as the A-CMA and P-CMA, respectively^16^. The observed differences in masticatory behavior may reflect the different projection pathways of these two areas. We have previously shown that an altered occlusal behavior by long-term feeding of soft diet affects the neuromuscular control of the jaw muscles induced by the P-CMA^19^. Anterograde tract-tracing studies of the CMA showed that the cortical projection from the A-CMA is denser in the inter-trigeminal region and reticular and is located ventrally to the trigeminal motor nucleus when compared to the projection from the P-CMA^16,20^. Moreover, the A-CMA partially overlaps with the orofacial motor area, which is necessary for fine motor control, somatic sensory integration, and cognitive motor movements^21^. Although the orofacial motor area encodes the tongue muscles, mouth, and jaw and plays an important role in orofacial motor functions, it remains unclear whether a change in occlusal behavior affects neuromuscular control induced by the A-CMA.

In this study, we sought to evaluate the developmental course of specific RJMs and examine the effect of long-term low occlusal loading on neuromuscular development of masticatory behaviors and the associated cortically-induced RJMs during growth. To this end, we examined both EMG activities of the jaw muscles and jaw movement trajectories that were elicited by repetitive stimulation of the A-CMA in rats fed soft diet. Findings were compared with a control group fed a normal diet across ages to examine whether low occlusal loading impedes masticatory-related neuromuscular control.

## Results

### Body weight

The body weights of the rats were monitored weekly throughout the experiment. There were no significant differences in the mean body weight between the two groups at any time point.

### EMG activities elicited by the A-CMA

**Figure 1:**
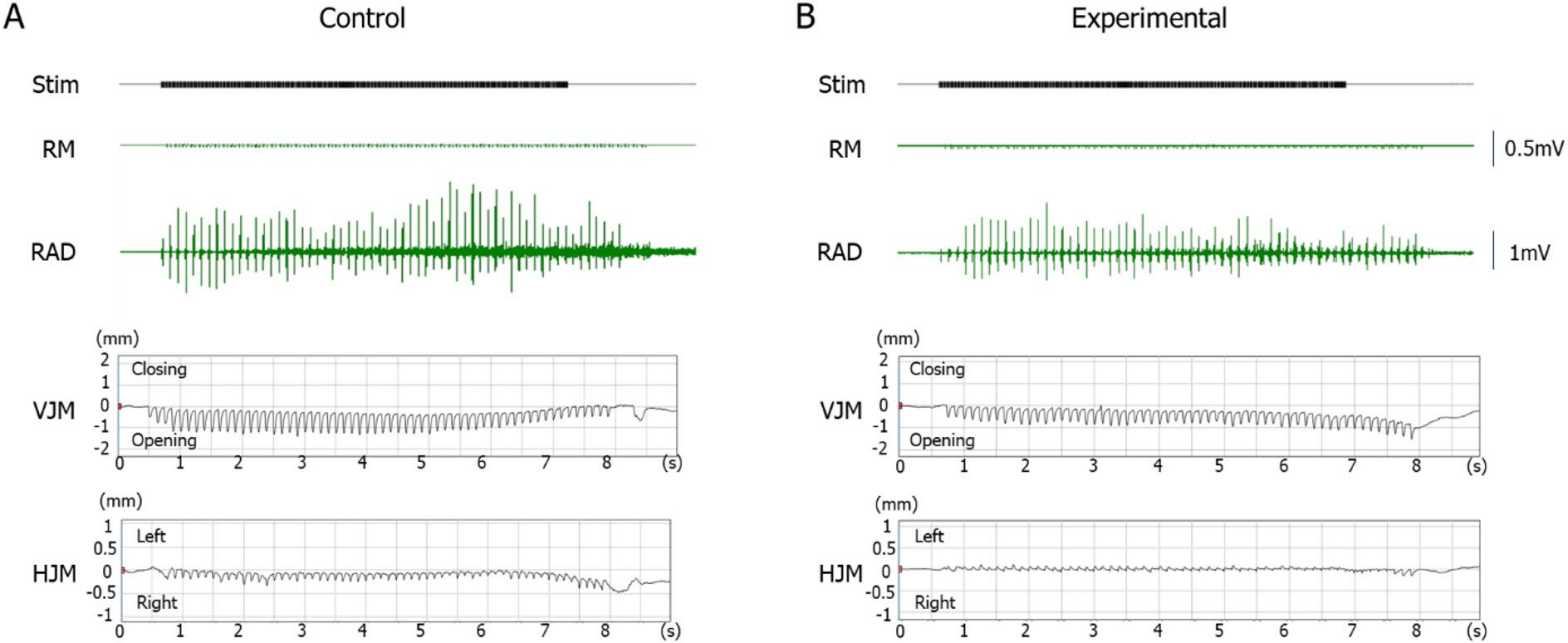
Typical example of electromyographic activity with jaw movement patterns elicited by electrical stimulation of the anterior part of the cortical masticatory area. Control (**A**) and experimental (**B**) groups at 11 weeks of age are shown. *Stim* electrical stimulation, *RM* right masseter muscle, *RAD* right anterior digastric muscle, *VJM* vertical jaw movements, *HJM* horizontal jaw movements.

Typical EMG activity with jaw movement patterns during stimulation are shown in Fig. 1, and the inset enlargement of both EMG activity and jaw movement trajectories of the two groups are shown in Supp. Fig 3. The jaw position was stable at rest, and the distance between the upper and lower incisors was approximately 2–2.5 mm before stimulation.

**Figure 2:**
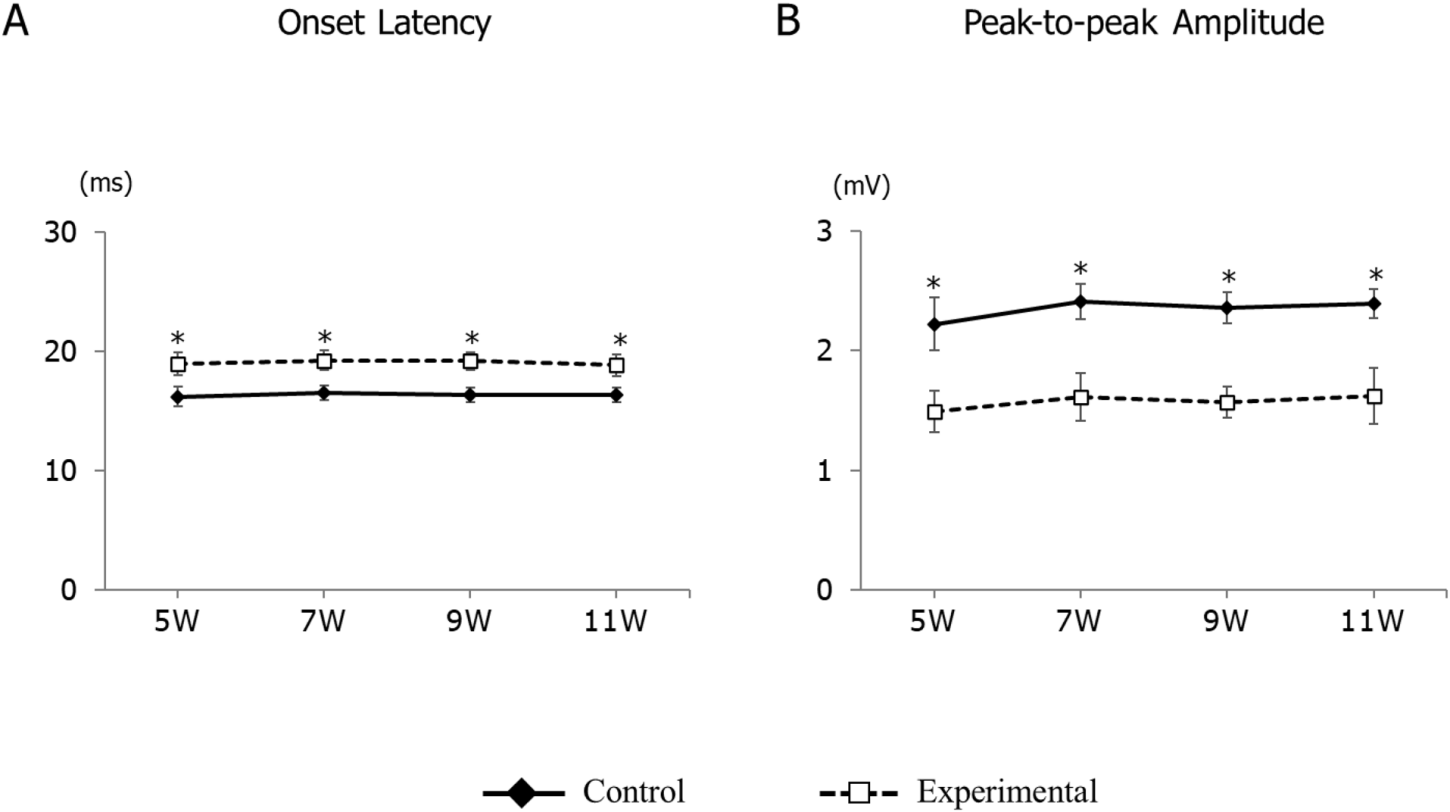
Longitudinal changes in electromyographic activity of onset latency (**A**) and peak-to-peak amplitude (**B**) in the control and experimental groups. Data are displayed as mean ± standard deviation. *p < 0.05 denotes significant differences between the control and experimental groups.

During repetitive electrical stimulation of the A-CMA, we found elicited EMG activities in the anterior digastric muscles; however, no activity was found in the masseter muscles in any of the experiments. Compared to that of the control group, the onset latency of the anterior digastric was significantly longer in the experimental group at each recording age; however, there were no significant differences in the intra-group comparisons at weeks 5, 7, 9, and 11 (Fig. 2A). The peak-to-peak amplitude was significantly smaller in the experimental group compared to that in the control group at each age. In contrast, in intra-group comparisons, the peak-to-peak amplitude showed no significant difference between the two groups at week 5, 7, 9, and 11 (Fig. 2B).

**Figure 3:**
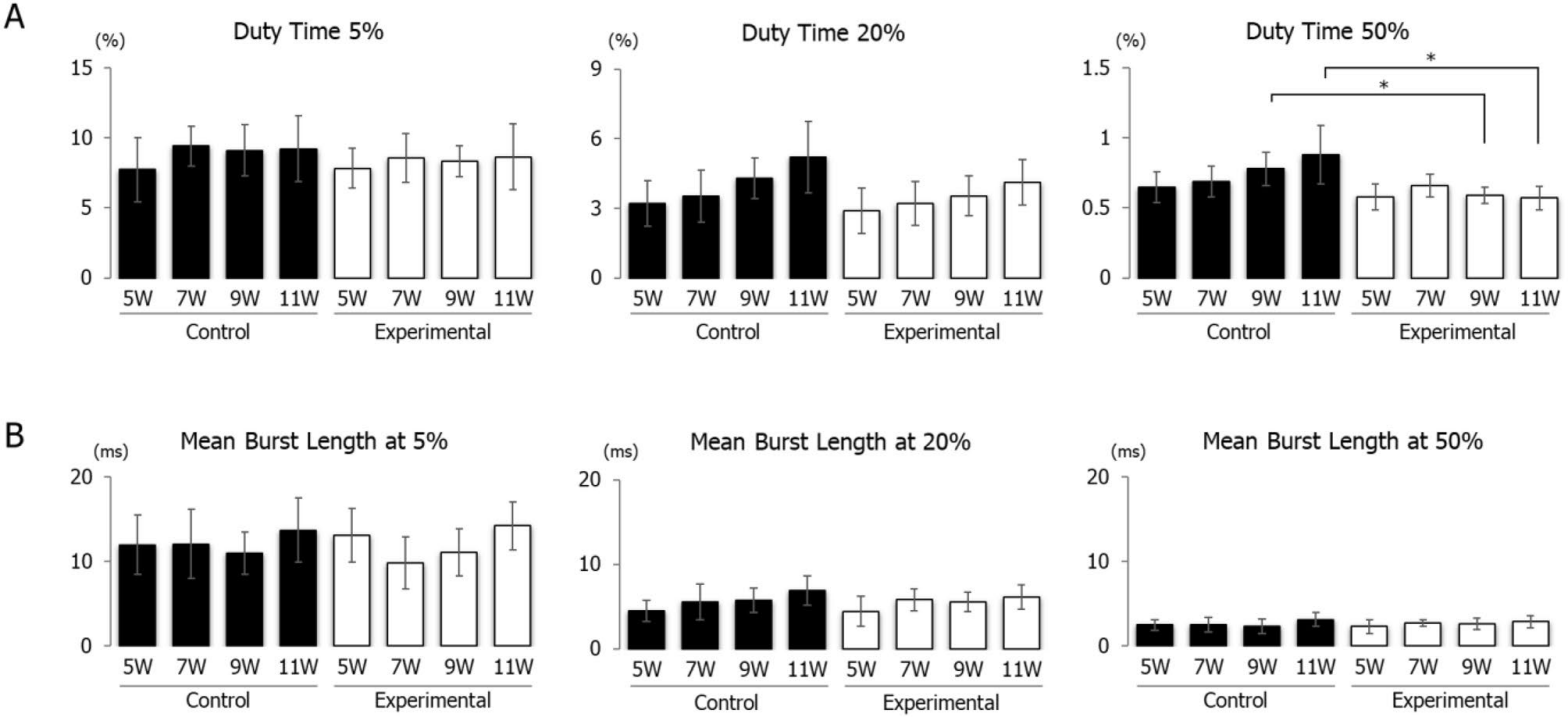
Longitudinal changes in duty time (**A**) and mean burst length (**B**) in the control and experimental groups. Values exceeding 5%, 20%, and 50% activity levels of the peak electromyographic activity are shown. Data are displayed as mean ± standard deviation. *p < 0.05 denotes significant differences between the control and experimental groups.

Regarding the peak EMG activity of the anterior digastric muscle, there was a significant difference in the duty times at levels exceeding 50% of peak EMG activity between control and experimental groups at weeks 9 and 11, whereas there was no significant difference in other ages. An intra-group comparison across ages revealed no significant differences in duty time exceeding 5, 20, and 50% activity levels in the control and experimental groups (Fig. 3A). The muscle burst length at 5, 20, 50% activity levels was not significantly different between the control and experiment groups at any age. An intra-group comparison among ages also revealed no significant differences in muscle burst length exceeding 5, 20, and 50% activity levels at any age (Fig. 3B).

**Figure 4:**
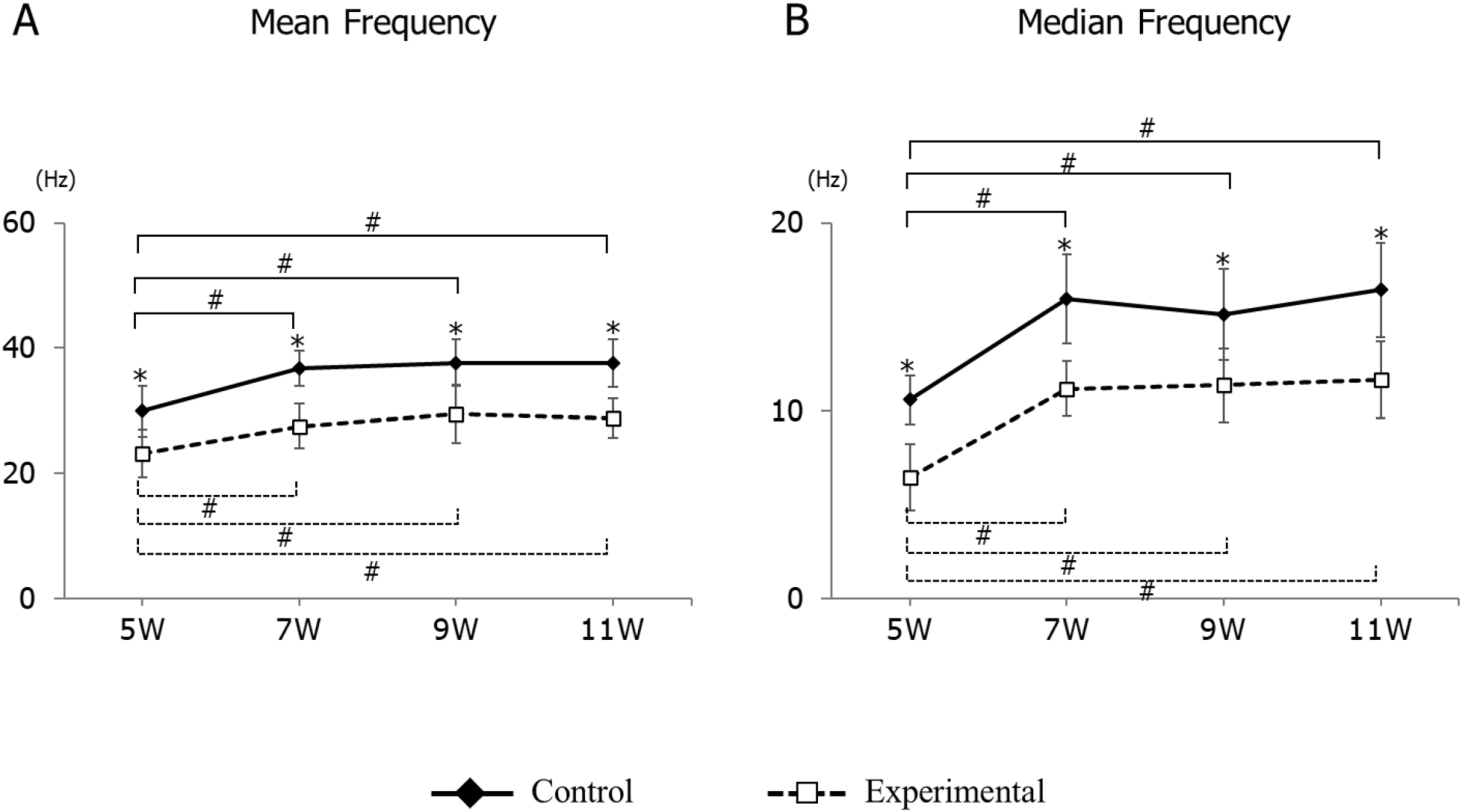
Longitudinal changes in mean frequency (**A**) and median frequency (**B**) of electromyographic activity in the control and experimental groups. Data are displayed as mean ± standard deviation. *p < 0.05 denotes significant differences between control and experimental groups. ^#^p < 0.05 denotes significant differences between different ages within the same group.

In the power spectrum analysis of the EMG signal, the average mean frequency in the experimental group was significantly smaller than in the control group at each age. An intra-group comparison between ages showed that the mean frequency at 5 weeks was significantly smaller than at 7, 9, and 11 weeks of age, while there were no significant differences between weeks 7, 9, and 11 in either the control or experimental groups (Fig. 4A). Likewise, the median frequency in the experimental group was significantly smaller than in the control group at each age. An intra-group comparison between ages showed that the median frequency at 5 weeks was significantly smaller than at 7, 9, and 11 weeks of age, while there were no significant differences between weeks 7, 9, and 11 (Fig. 4B).

### RJMs trajectories elicited by the A-CMA

**Figure 5:**
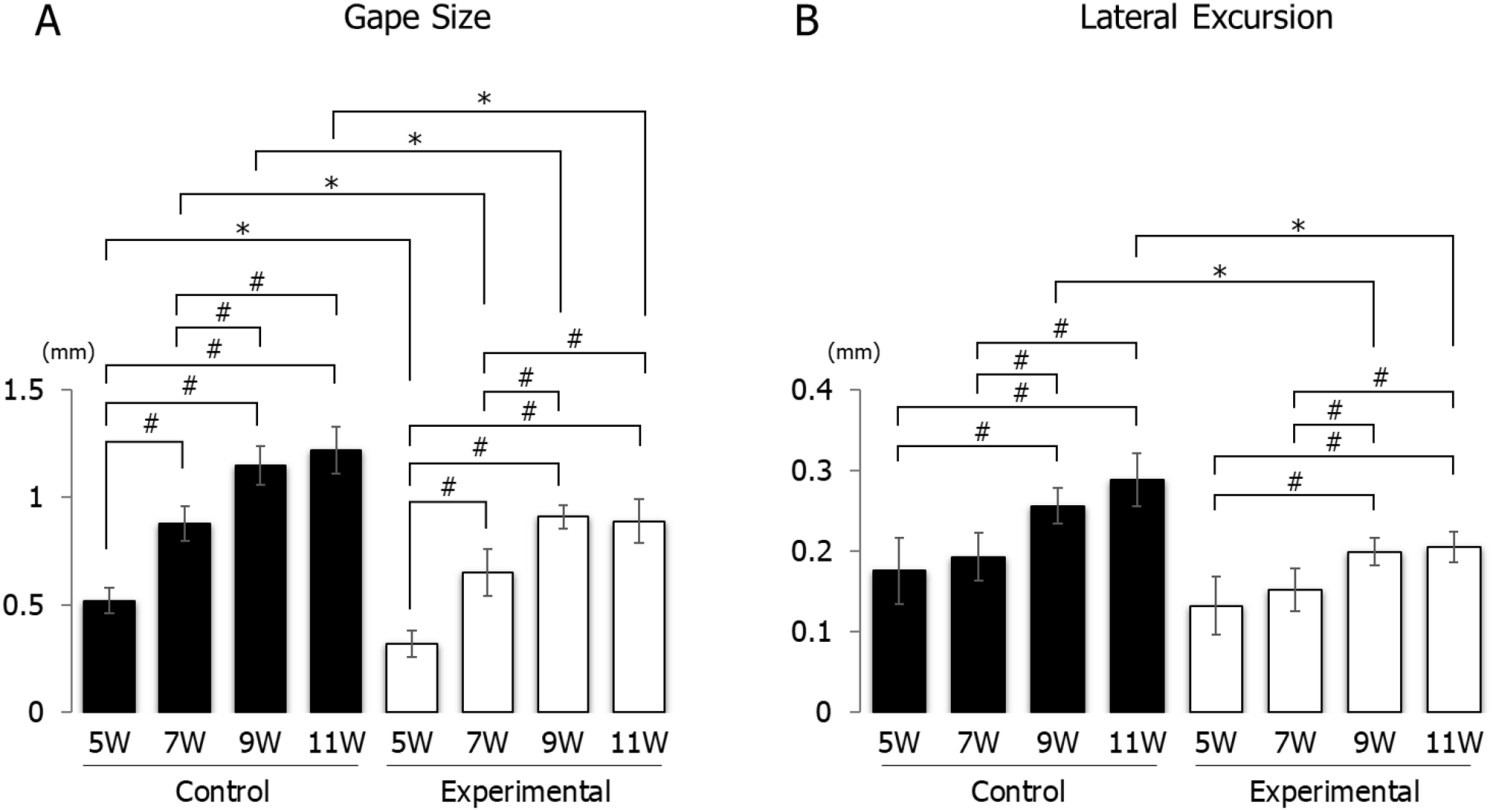
Longitudinal changes in jaw movements during stimulation. Gape size (**A**) and lateral excursion (**B**) are shown. Data are displayed as mean ± standard deviation. *p < 0.05 denotes significant differences between control and experimental groups. ^#^p < 0.05 denotes significant differences between different ages within the same group.

During sustained stimulation, the gape size of jaw movement in the experimental group was significantly smaller than that in the control group at each recording age. When compared to the control group, the gape size in the control group at 9 and 11 weeks was significantly larger than at 5 and 7 weeks, and the gape size at 7 weeks was significantly larger than at 5 weeks; however, there was no significant difference between weeks 9 and 11. Likewise, the gape size in the experimental group at 9 and 11 weeks was significantly larger than those at 5 and 7 weeks, and at 7 weeks was significantly larger than that at 5 weeks; however, there was no significant difference between weeks 9 and 11 (Fig. 5A).

Lateral excursion was significantly smaller in the experimental group than that in the control group at weeks 9 and 11; however, there was no significant difference between groups at weeks 5 and 7. An intra-group comparison in the control group showed that lateral excursion at weeks 9 and 11 was significantly larger than at weeks 5 and 7, while there were no differences between weeks 5 and 7, and weeks 9 and 11. A similar trend was found in the experimental group (Fig. 5B).

**Figure 6:**
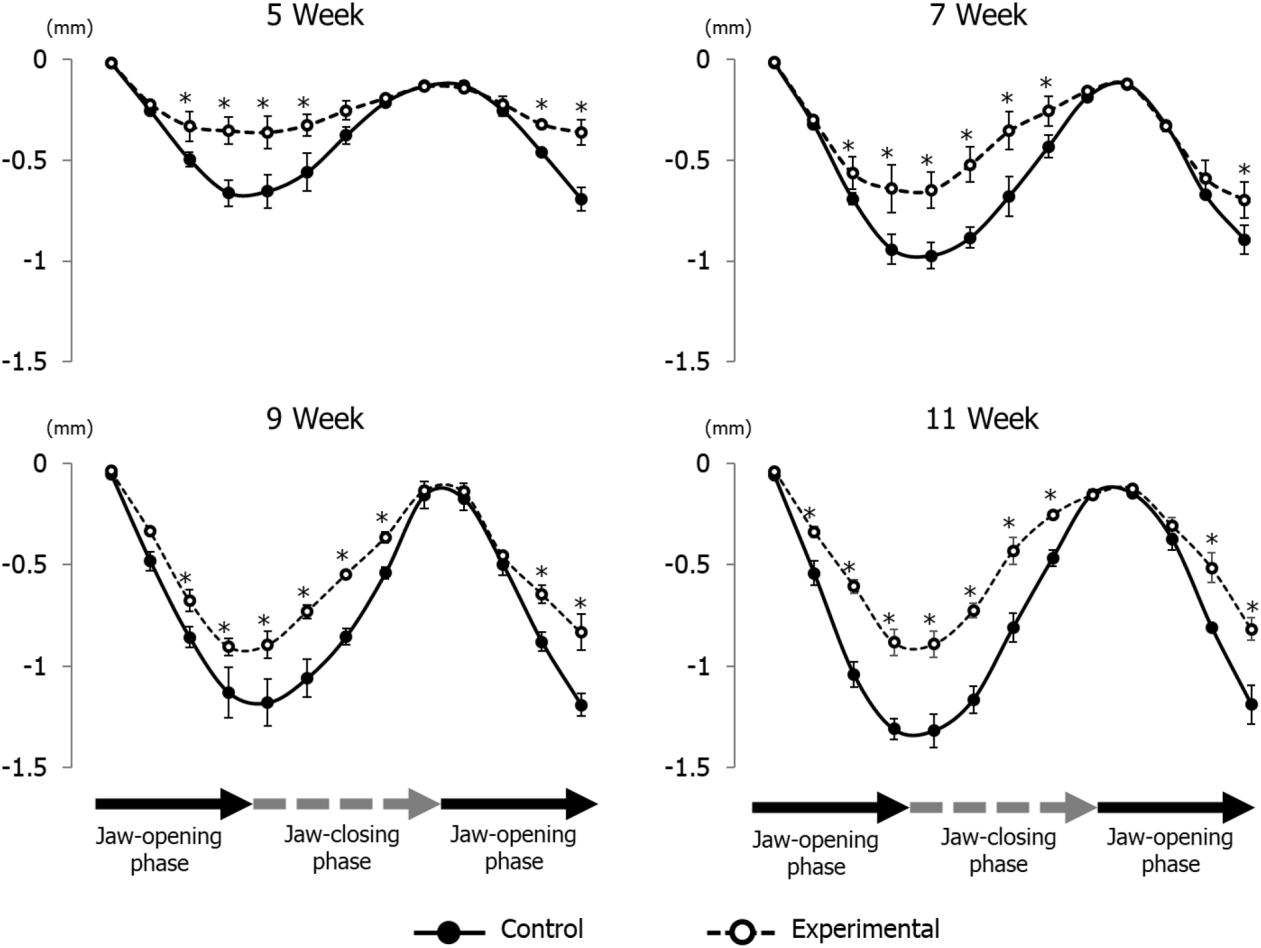
Longitudinal comparison of the path of vertical jaw movement between the control and experimental groups during jaw-opening and closing phases. The 13 points mark the 10 ms intervals throughout the masticatory cycle. Data are displayed as mean ± standard deviation. *p < 0.05 denotes significant differences between the control and experimental groups.

**Figure 7:**
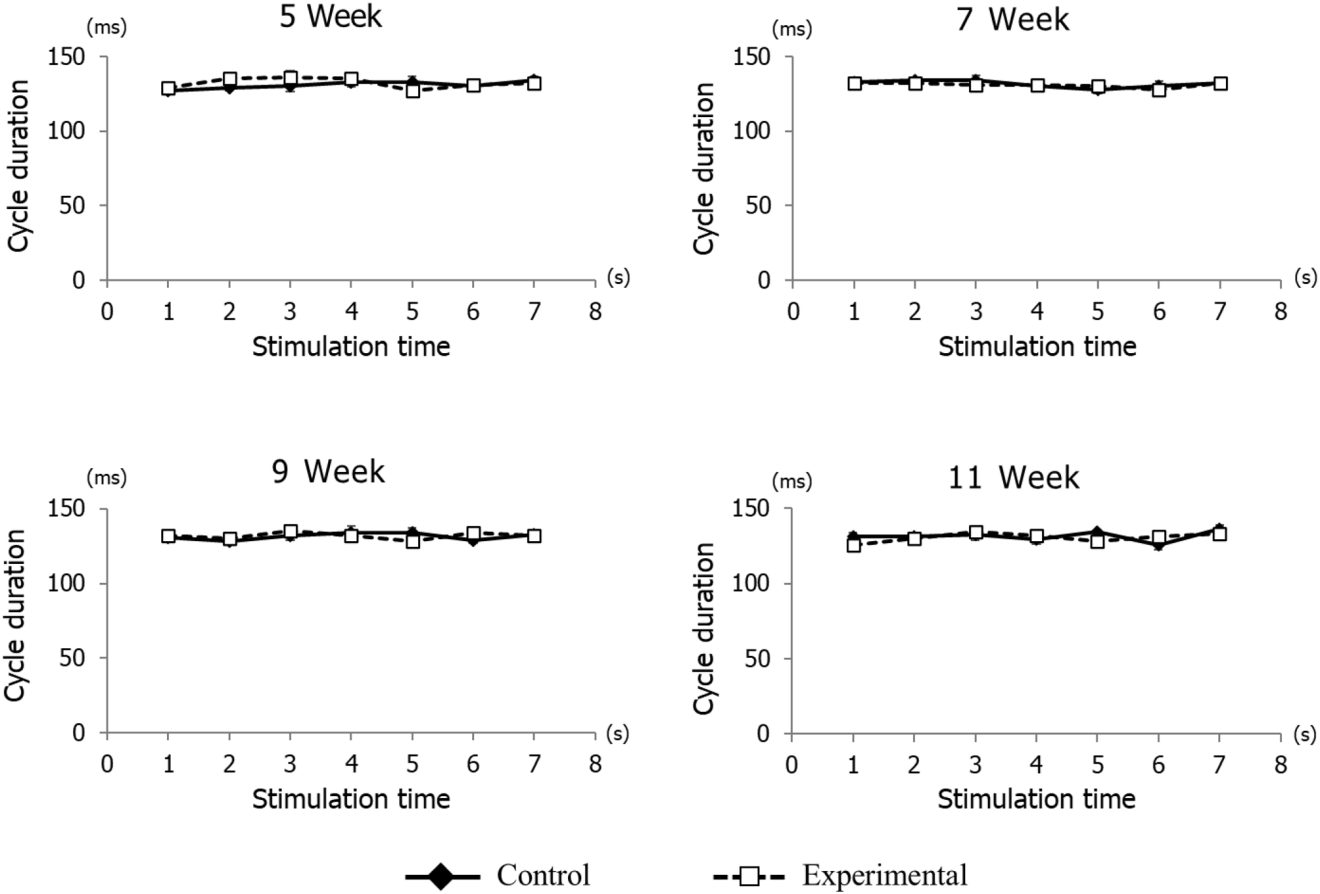
Rhythm of jaw movement cycle duration during repetitive stimulation at each time point. Rhythm of cycle durations was not significantly different in either group during repetitive stimulation per second.

**Figure 8:**
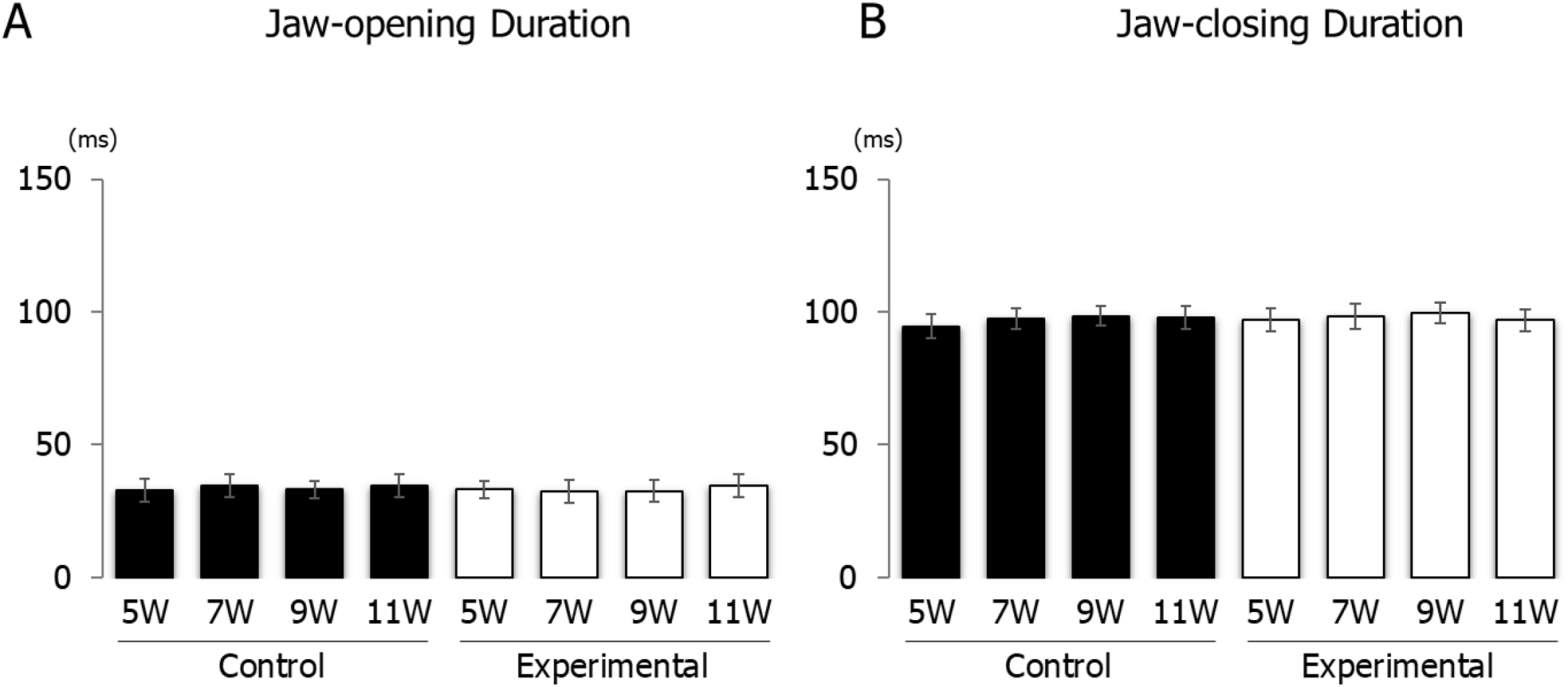
Duration of jaw movement phases during electrical stimulation. Jaw-opening duration (**A**) and jaw-closing duration (**B**) of control and experimental groups.

The vertical jaw movement paths showed a similar pattern between groups at each recording age. However, the traces at maximum opening position, referred to as gape size, showed significant differences between groups at each recording age (Fig. 6). Comparing jaw movement rhythm showed no significant differences between control and experimental groups during stimulation (Fig. 7). With regard to the duration of jaw movements, there were no significant intra-group or between group differences in jaw-opening (Fig. 8A) and jaw-closing (Fig. 8B) durations at each age.

## Discussion

We found that the long-train A-CMA stimulation induced RJMs in both groups. A previous study has suggested that electrical stimulation of the P-CMA induces vigorous saliva secretion with masticatory like rhythmical jaw movements, whilst A-CMA stimulation induces small and simple rhythmical jaw movements^22^. Repetitive electrical stimulation applied to the A-CMA appears to activate the masticatory CPG in the brainstem, which induces RJMs. The stimulus intensity required for CMA stimulation to elicit RJMs in rats has been shown to vary between 50 and 300 µA, and it has been noted that the threshold for RJMs evoked from the A-CMA is smaller compared to that of the posterior region^16,23^. We used a constant current strength (120 µA) to compare the cortically-elicited RJMs and found that long-term alteration of the masticatory load at an early age affected the developmental course of jaw movements and the neuromuscular behavior of the masticatory muscle.

The corticofugal projection from the A-CMA reaches the contralateral side of the masticatory CPG via the cerebral peduncle and pyramidal tract^24^. In contrast, the descending impulses from the A-CMA activate neurons in the superior colliculus that project to the intermediate reticular formation as well as the parvocellular reticular formation that innervates the jaw-opening and jaw-closing motoneurons in the masticatory CPG as well as the trigeminal motoneurons^25^. Moreover, EMG activity in our study was only demonstrated in the anterior digastric muscle with a vertical pattern of RJMs, while no activity of the masseter muscle was observed during stimulation of the A-CMA. According to a cytoarchitecture study in the cortical areas, the agranular cortex induces RJMs through activity of the anterior digastric muscle, whereas the granular area induces RJMs through alternating activity of the masseter and anterior digastric muscles^26^. Moreover, it is possible to induce a vertical pattern of RJMs by microstimulation applied to the anterior agranular field of the cortical area^26^. Thus, our findings support a vertical pattern of RJMs, determined by jaw muscle EMG activity and jaw movements, which displayed the features of the RJMs elicited by stimulation of the A-CMA.

In our study, the onset latency and peak-to-peak amplitude of the anterior digastric muscle remained stable throughout the experiment in both groups. Latency reflects the conduction velocity of nerve fibers and is affected by the state of myelination of the nerve, whereas the peak-to-peak amplitude assess voluntary contraction force regulated by the muscle fibers and motor units during muscle contraction^27,28^. Cortical projections from A-CMA are involved in voluntary control of the masticatory muscles via the corticobulbar fibers to trigeminal motoneuron pools in the brainstem^24^. The growth maturation of craniofacial structures including oral mechanoreceptors (e.g., periodontal and temporomandibular joint mechanoreceptors) and trigeminal neurons begin by the age of 5 weeks, and reaches a plateau around 11 weeks in rats^29–31^. The long latency appears in the young rats immediately before the cessation of the weaning period (i.e., 3 weeks of age); however, it disappears at 5 weeks of age^32^, and the anterior digastric muscle attains its specific adult fiber-type profile at approximately 6 weeks^33^. Our study also indicated that the onset latency and peak-to-peak amplitude of the anterior digastric muscle are not affected by aging throughout the experimental period.

We have also identified a constant peak EMG throughout the experimental period in both groups. Measurement of the duty time of the peak EMG is important not only for the assessment of the activation of jaw muscles but also for the development of muscle function during growth. The duty time of the peak EMG is determined by the total duration of muscle activity at 5%, 20%, and 50% during the recording period, and the development of jaw muscle activation remains stable during the growing stage^34^. For example, the duty time of the anterior digastric muscle remains largely unchanged from the suckling stage to chewing stage and then throughout postnatal development. The duty time study also reported that age-related changes do not affect the overall duty time of the jaw muscle^34^, resulting in stable daily muscle activity during aging. The mean burst length did not differ throughout the experiment in either group. Previous studies on jaw muscles have shown that the amount of jaw muscle activation shows relatively few changes during development, and the cycle duration of chewing behavior remains unchanged until postnatal age^34,35^. In addition, during stimulated muscle contractions, the duration of jaw muscle contraction is controlled by the masticatory CPG in response to repetitive electrical stimulation of the CMA^36,37^. Thus, this suggests that the masticatory CPG maintains muscle contractions during stimulation of the CMA, maintaining a stable muscle burst length at different ages.

A power spectral analysis revealed that both the mean and median frequencies increased between  5 and 7 weeks, and plateued  at 9 and 11 weeks in both the control and experimental group. A frequency analysis assessing how the EMG signal varies with time has been used to monitor muscle fatigue during muscle contractions^38^. The contribution of muscle fibers to the varying EMG power spectrum size is associated with variations in the proportion of muscle fiber diameter. Moreover, the muscle geometry, including fiber diameter and muscle length, significantly affects the time-varying EMG spectrum^39^. Furthermore, a previous study has reported that the skeletal muscle fibers of rats undergo intense growth from 3 to 10 weeks of age, with an increase in the number and size of myofibers, and attain stable growth after 10 weeks^40^. This suggests that changes in the morphology of the muscle influence the frequencies of the EMG power spectrum.

This study identified a significant difference in onset latency between the control and experiment groups at all ages. The delayed onset latency was observed during soft diet feeding, which alters the oxidative capacity of jaw muscle fibers given less muscle usage, which results in immature peripheral sensory feedback^41–43^. The prolongation of the onset latency was supported by our previous study in which we found that the jaw muscle was evoked by stimulation of the P-CMA^19^, and this study also suggests that low occlusal loading not only affects the peripheral system but also alters the central response that is specifically elicited by stimulation of the A-CMA.

The peak-to-peak amplitude was significantly reduced in the experimental group after soft diet feeding, as in our previous study^19^. Animals feeding with soft diet showed a significant decrease in the cross-sectional area of the type 2 fibers of the jaw-opening muscle^35,44^. It has been suggested that changes in occlusal loading affects the capacity of modulatory sensory feedback from the masticatory CPGs^36^. In additions, the oral receptors, including periodontal afferents and muscle fibers, convey the major input sources for the masticatory CPGs. For example, diminished activity of muscles and masticatory force appears during abolishing the signal information from the periodontal receptors and muscle spindle receptors^45^. Findings from our study revealed that occlusal hypofunction influenced burst generating neurons, resulting in decreased EMG activity of the muscle.

Our study also observed a significantly low duty time in muscle activity exceeding 50% at weeks 9 and 11, whereas the activities exceeding 5 and 20% were not significantly different between groups at any age. A report of the muscle activity in humans showed that more than 80% of the muscle activity exceeding 25% was observed during mastication^46^. Moreover, the duty time of the peak EMG exceeding 5% appeared to represent overall usage of muscle activity, whereas activity exceeding 50% was representative of forceful muscle recruitment in a previous stud^14^. During training with low occlusal loading, the amount of low muscle activity increased due to a slow-to-fast transition of the myosin heavy chain isoform, which resulted in a small amount of powerful muscle contractions^14,47^. Thus, it is likely that in our study, low occlusal loading induced by a soft diet did not affect the overall daily activity at lower activity levels; however, an increased amount of low muscle activity influenced higher activity levels, especially in forceful muscle constriction.

In this study, the average burst length was constant in every muscle activity exceeding 5%, 20%, and 50% at all recording ages. It has been reported that altering the masticatory load does not affect the burst length at any activity level^14^. Moreover, the duration of muscle contraction is mainly achieved by modulating the response from the masticatory CPGs, which results in a relatively constant frequency of burst length in a muscle^36,47^. Therefore, it is likely that the A-CMA stimulation induces excitatory activation of jaw muscles via masticatory CPGs, yielding a stable duration of burst length during stimulation of muscle contractions.

Regarding the time frequency analysis of the muscles, both the mean and medium frequencies of the muscle decreased after soft diet feeding. The mean frequency determines changes in muscle fatigue over time, whereas the median frequency measures two equal halves of the spectrum during muscle contraction^38^. A previous study found that a smaller frequency in EMG spectrogram is associated with decreased muscle fibers^48^. Moreover, decreased masticatory loading occurs when the muscle fibers transition from type 1 to type 2 fibers, followed by a reduction in muscle fiber size^49^. Thus, it is likely that the shifting of muscle fibers due to the low occlusal loading effects altered the muscle fiber size, thereby causing muscle fatigue during contraction.

The jaw movements in our study showed simple vertical RJMs with time-locked activity of the anterior digastric muscle. Many studies on humans and animals have found that masticatory hypofunction induced by changing food consistency affects masticatory performance^50–52^. It has also been reported that being raised on a soft diet during early life affects the functional activities of the jaw and tongue muscles of rats^43^. Previous studies have suggested that occlusal hypofunction significantly decreased the number of muscle fibers and mitochondria per unit in masticatory muscles in growing rats^14,53^. Interestingly, in our study, the gape size during vertical RJMs in soft diet fed rats appears below the normal level throughout the experimental periods. It is likely that masticatory hypofunction due to low occlusal loading affects the activities of mechanoreceptors in oro-facial regions, and impairs the functional maturation of jaw movements.

The patterns of masticatory behavior in this study were similar between the two groups across recording ages. A diet-related study has shown that changing to a soft diet alters the motor unit activity of muscles due to an impairment of the morphological and metabolic properties of the muscle, but no changes in soma diameter and enzymatic activity of motoneurons have been found^54^. During stimulation of the A-CMA, a similar pattern of jaw movements was observed in both groups, except for the gape size. At week 5, the jaw movement paths showed significant difference around the maximum opening position, and this difference was larger 2 weeks after being fed a soft diet. The jaw movement paths in maximum gape size at week 7, 9, and 11 showed significantly reduced in the rats with low occlusal loading, suggesting that long-term feeding the soft diet during the developmental period had a larger effect on masticatory performance, resulting in altered jaw-opening position through the experimental period.

Masticatory CPGs play a role in the timing and sequence of RJMs. During electrical stimulation, descending projections from the A-CMA reach the lateral pontomedullary reticular formation, generating the repetitive bursting of neurons for RJMs^55^. It has been reported that these bursting neurons are detected as early as postnatal days 9–12, and rapidly increase in activity up until postnatal day 14, after which they remain constant^56,57^. We found no significant differences in the duration parameters as a function of age, including jaw-opening and jaw-closing duration, in either the control or experimental group. This finding demonstrates that masticatory CPGs maintain the cycle duration of RJMs that resembles the duration of the stimulus interval to the A-CMA.

Changes in masticatory function are thought to reflect the motor organization within the somatosensory cortex. A change in occlusal contact such as molar tooth extraction or dental trimming results in a decreased jaw and tongue motor representation^10^, and the diminished action of peripheral sensory innervation by transection of the lingual nerve significantly decreases tongue motor representations within the motor cortex^58^. Masticatory CPGs mainly regulate the rhythmic trigeminal activity of masticatory movements, while peripheral sensory information modulates the central motor command^25^. Neural signals from the A-CMA activate the brainstem masticatory CPGs within the reticular formation and trigeminal motor nuclei, which induces various trigeminal actions to orofacial regions. In our study, vertical RJMs with clusters of sub-burs time-locked activity of the anterior digastric muscle were observed during repetitive stimulation of the A-CMA, and these cortically-elicited RJMs and neuromuscular responses were impaired in the occlusal hypofunction group. Therefore, the findings in our study show that consuming a soft diet during early development inhibits the motor performance of higher brain centers, resulting in delayed neuromuscular behaviors from the jaw sensorimotor cortex during stimulation of the A-CMA.

This study illustrated that masticatory hypofunction, from the long-term consumption of a soft diet during development, impairs the A-CMA-elicited masticatory neuromuscular behaviors, and these impaired behaviors do not return to normal until adulthood. Previous intracranial microstimulation studies demonstrate that stimulation of the CMA, A-CMA and P-CMA generate different masticatory behaviors^16^. We also found that for the A-CMA, the maximum gape size of jaw movement behavior appeared at a current strength of 120 µA. In contrast, in our previous study^19^, the P-CMA induced the maximum gape size of jaw movement at a current strength of 180 µA. While the characteristics of jaw movement elicited by stimulation of the A-CMA and P-CMA did not differ between the studies, the amplitude of the maximum gape size and the lateral excursion varied depending on the stimulation site. The jaw movements elicited by stimulation of the A-CMA began with jaw opening followed by rhythmical movements of simple opening and closing behavior. Moreover, projection of the oral sensory signals to the insula cortex, which partially overlaps with the P-CMA in rats, suggests that sensory deprivation from the oral region might impact the P-CMA more than it does the A-CMA^22^. However, the A-CMA showed that low occlusal loading after weaning impaired the EMG behaviors of the jaw muscles and masticatory movements. Therefore, oral sensory deprivation in association with low occlusal loading in our study may induce neuroplastic changes within the motor representation of the A-CMA, and that sensory inputs from the oral regions modulate neuromuscular behaviors of the CMA.

## Conclusions

Low occlusal loading during growth induces alterations in EMG activity and behavior of the anterior digastric muscle and produces changes in jaw movements that are mediated by the projection from the A-CMA. Jaw movement regulation and the neuromuscular control of jaw muscles play an important role in masticatory behavior in early life, and each part of the CMA has a significant influence on regulation of masticatory behavior. Therefore, masticatory hypofunction during growth should be monitored and corrected as soon as possible to avoid any negative effects on craniofacial development and physiological behavior.

## Methods

### Animal model and surgical preparation

Sixty-four 2-week-old male albino Wistar rats were used in this study. All young pups were examined and confirmed to still be weaning to prevent any experience of chewing a solid diet from being included in the experimental group. All experiment procedures were performed in accordance with relevant guidelines and regulations, and approved by the Institutional Animal Care and Use Committee (A2017-135A and A2018-028A) in compliance with the Animal Care Standards of Tokyo Medical and Dental University (TMDU). All procedures including animal handling, housing, stimulation and surgical procedures were performed in accordance with the ARRIVE guidelines.

The rats were randomly divided into either the control or experimental group (n = 32 each). The control group was fed ordinary chow pellet (CE-2, CLEA Inc, Tokyo, Japan), while the experimental group was fed CE-2 powder pellet (< 0.02 mm diameter) until 11 weeks of age. Food and water were freely accessible at all times, and both groups were weighed weekly throughout the entire experimental period. All rats underwent the experimental procedure at 5, 7, 9, or 11 weeks of age (n = 8 per group per time point). Ketamine-HCl (100 mg/kg, intraperitoneal; IP) injection was administrated initially for the craniotomy and EMG electrode insertion. Supplementary doses were injected whenever necessary to maintain a constant level of anesthesia according to checks of vibrissa movements, the pinch-withdrawal, and corneal reflexes throughout the experiments. The local anesthetic lidocaine hydrochloride (2%) was injected into the subcutaneous space below the planned surgical areas. Body temperature was maintained at 37–38 °C using a thermo-regulated heating pad.

A midline incision was made along the neck from the mandible on the ventral surface to the rostral portion to expose the right side of masseter (jaw-closer) and anterior digastric (jaw-opener) muscles to record EMG activity from the jaw muscles^19,22,23^. Bipolar EMG electrodes (40 gauge, single-stranded, Teflon insulated stainless-steel wires, 2 mm inter-electrode distance) were then inserted into the muscles to record EMG activity^19,22,23^. A part of the left frontal and parietal bones were drilled using a dental bar to expose the outer surface of the masticatory cortex to stimulate the cerebral cortex, and the dura mater was covered with paraffin liquid oil (37 °C). The rat was then secured in a stereotaxic apparatus (models SN-2 and Sm-15 M; Narishige Scientific Instruments, Tokyo, Japan). A fine glass-insulated tungsten microelectrode (shaft diameter 100 µm, impedance 1–3 MΩ at 1 kHz; Unique Medical, Tokyo, Japan) was inserted vertically into the masticatory area of the cerebral cortex. Electrical stimulation (0.5 ms duration, 20 Hz, 120 µA, 7 s) was applied to the left A-CMA (3–4 mm anterior, 2–3.5 mm lateral to bregma, 2–3.5 mm deep from cortical surface). A reference electrode was attached to the exposed neck muscle. RJMs were determined by mandibular movements and rhythmic bursts of the anterior digastric or masseter EMG activities. Three trials of stimulation were performed at each stimulation site.

### Recording of jaw movements and EMG activity

For recording sessions, a wire (0.7 mm thick) attached to a marker was placed between the lower incisors attached with dental resin. A digital high-speed HAS-U1M camera (DITECT Corp, Tokyo, Japan) was set directly in front of the marker to detect jaw movements. During stimulation, the jaw movements were videotaped, and a 2D motion analysis system software (Dipp-motion V, DITECT Cop, Tokyo, Japan) was used to refine the marker position of the jaw movements as described in our previous study^19^. EMG activity signals were filtered and amplified using a multichannel amplifier (MEG-6108; Nihon kohden, Tokyo, Japan; 1000× gain, bandpass 0.3–3 kHz), and all EMG waveforms were rectified and averaged. Data were analyzed offline using the CED 1401 interface and Spike2 software for Windows, version 5.21 (Cambridge Electronic Design, Cambridge, UK). EMG responses of each muscle were analyzed in a fast sweep. To analyze the specific characteristics of EMG, the sonogram was calculated based on the peak of root-mean squared EMG activity and the power spectrum was displayed using a fast Fourier transform.

The RJMs and EMG activity recordings were stored in a computer disk. Jaw movement patterns during stimulation were observed in the frontal plane. A list of the parameters in this paper, refer to Supp. Table 1. The mean values of data for each parameter were measured for ten chewing cycles, and the average was calculated. The EMG bursts were identified when the rectified EMG exceeded the mean by 2 standard deviations (SDs), and the duty time and burst length were defined based on the number and lengths of bursts with amplitudes exceeding 5%, 20%, and 50% of the peak-EMG activity (Supp. Fig. 1). All values are expressed as mean ± SD.

### Histological identification of electrode positioning

After the experiment was completed, the rats remained on the stereotaxic apparatus and were anesthetized with Ketamine-HCl (20 mg/kg, IP); we created the electrical lesions by passing currents (30 µA for 20 s) through the stimulating electrode at the same area to check the location of the electrode tips. The rats were then deeply anesthetized and perfused with 100 ml of phosphate-buffered saline (PBS; pH 7.4) through the left cardiac ventricle followed by 300 ml of fixative solution of 4% paraformaldehyde. Serial coronal sections of the brain (50 µm thick) were cut and counterstained with hematoxylin–eosin stain. The locations of the electrode tips were confirmed under a light microscope and verified using the reference^59^ (Supp. Fig. 2).

### Statistical analysis

Significant differences between control and experimental groups were determined using an unpaired t-test, and repeated-measures multivariate analysis for inter- and intra-group comparisons of jaw movement trajectories and EMG activities. Simple post hoc tests using the Sidak adjustment were performed for multiple comparisons. Statistical analysis was performed with SPSS for Windows, version 23 (SPSS Inc., Chicago, IL), and p values < 0.05 were considered to be statistically significant.

## Supplementary Information

Supplementary Information.

## Data Availability

The data that support the findings of this study are available upon request from the corresponding author.

## References

[1] Kurnikova A, Moore JD, Liao SM, Deschênes M, Kleinfeld D (2017). Coordination of orofacial motor actions into exploratory behavior by rat. Curr. Biol..

[2] Ingram TGJ, Solomon JP, Westwood DA, Boe SG (2019). Movement related sensory feedback is not necessary for learning to execute a motor skill. Behav. Brain Res..

[3] Maznychenko AV (2019). Activity of motor units in human elbow flexor and extensor muscles during task-dependent unloading. Neurophysiology.

[4] Canu MH, Darnaudery M, Falempin M, Maccari S, Viltart O (2007). Effect of hindlimb unloading on motor activity in adult rats: Impact of prenatal stress. Behav. Neurosci..

[5] Neafsey EJ (1986). The organization of the rat motor cortex: A microstimulation mapping study. Brain Res..

[6] Yao D (2002). Neuronal activity patterns in primate primary motor cortex related to trained or semiautomatic jaw and tongue movements. J. Neurophysiol..

[7] Abe Y (2017). Unilateral nasal obstruction affects motor representation development within the face primary motor cortex in growing rats. J. Appl. Physiol..

[8] Koecklin KHU (2015). Effect of unilateral nasal obstruction on tongue protrusion forces in growing rats. J. Appl. Physiol..

[9] Sood M, Lee JC, Avivi-Arber L, Bhatt P, Sessle BJ (2015). Neuroplastic changes in the sensorimotor cortex associated with orthodontic tooth movement in rats. J. Comp. Neurol..

[10] Avivi-Arber L, Lee JC, Sessle BJ (2015). Dental occlusal changes induce motor cortex neuroplasticity. J. Dent. Res..

[11] Kato C (2012). Increased occlusal vertical dimension induces cortical plasticity in the rat face primary motor cortex. Behav. Brain Res..

[12] Kono K, Tanikawa C, Yanagita T, Kamioka H, Yamashiro T (2017). A novel method to detect 3D mandibular changes related to soft-diet feeding. Front. Physiol..

[13] Tanaka E (2007). Effect of food consistency on the degree of mineralization in the rat mandible. Ann. Biomed. Eng..

[14] Kawai N (2010). Adaptation of rat jaw muscle fibers in postnatal development with a different food consistency: an immunohistochemical and electromyographic study. J. Anat..

[15] Anegawa E (2015). Chronic powder diet after weaning induces sleep, behavioral, neuroanatomical, and neurophysiological changes in mice. PLoS ONE.

[16] Sasamoto K, Zhang G, Iwasaki M (1990). Two types of rhythmical jaw movements evoked by stimulation of the rat cortex. Jpn. J. Physiol.

[17] Lund JP, Sasamoto K, Murakami T, Olsson KA (1984). Analysis of rhythmical jaw movements produced by electrical stimulation of motor-sensory cortex of rabbits. J. Neurophysiol..

[18] Liu ZJ (1993). Coordination of cortically induced rhythmic jaw and tongue movements in the rabbit. J. Neurophysiol..

[19] Aung PT (2020). Functional analysis of rhythmic jaw movements evoked by electrical stimulation of the cortical masticatory area during low occlusal loading in growing rats. Front. Physiol..

[20] Zhang GX, Sasamoto K (1990). Projections of two separate cortical areas for rhythmical jaw movements in the rat. Brain Res. Bull..

[21] Sanes JN, Donoghue JP (2000). Plasticity and primary motor cortex. Annu. Rev. Neurosci..

[22] Maeda N (2014). Differential involvement of two cortical masticatory areas in submandibular salivary secretion in rats. Brain Res..

[23] Satoh Y, Ishizuka K, Murakami T (2006). Modulation of cortically induced rhythmical jaw movements by stimulation of the red nucleus in the rat. Brain Res..

[24] Yoshida A (2009). Corticofugal projections to trigeminal motoneurons innervating antagonistic jaw muscles in rats as demonstrated by anterograde and retrograde tract tracing. J. Comp. Neurol..

[25] Nakamura Y, Katakura N (1995). Generation of masticatory rhythm in the brainstem. Neurosci. Res..

[26] Isogai F (2012). Cortical area inducing chewing-like rhythmical jaw movements and its connections with thalamic nuclei in guinea pigs. Neurosci. Res..

[27] Lizarraga I, Chambers JP, Johnson CB (2007). Developmental changes in threshold, conduction velocity, and depressive action of lignocaine on dorsal root potentials from neonatal rats are associated with maturation of myelination. Can. J. Physiol. Pharmacol..

[28] Kamen G, Gabriel D (2010). Essentials of Electromyography.

[29] Hiranuma M (2013). Effects of a liquid diet on the response properties of temporomandibular joint nociceptive neurons in the trigeminal subnucleus caudalis of growing rats. Orthod. Craniofac. Res..

[30] Abed GS, Buschang PH, Taylor R, Hinton RJ (2007). Maturational and functional related differences in rat craniofacial growth. Arch. Oral Biol..

[31] Nasution FH, Toda K, Soma K (2002). Functional maturation of periodontal mechanoreceptors during development in rats. Brain Res. Dev. Brain Res..

[32] Thexton AJ, German RZ, Crompton AW (1995). Latency of the jaw-opening reflex in the premature, preweaning and adolescent pig (hanford strain miniature pig, *Sus scrofa*). Arch. Oral Biol..

[33] Hurov J (1992). Metabolic transitions in rat jaw muscles during postnatal development. J. Craniofac. Genet. Dev. Biol..

[34] van Wessel T, Langenbach GE, Brugman P, Korfage JA, van Eijden TM (2006). Daily activity of the rabbit jaw muscles during early postnatal development. Neuroscience.

[35] Langenbach GE, van Wessel T, Brugman P, van Eijden TM (2004). Variation in daily masticatory muscle activity in the rabbit. J. Dent. Res..

[36] Lund JP, Kolta A (2006). Generation of the central masticatory pattern and its modification by sensory feedback. Dysphagia.

[37] Lund JP (1991). Mastication and its control by the brain stem. Crit. Rev. Oral Biol. Med. Off. Publ. Am. Assoc. Oral Biol..

[38] Cifrek M, Medved V, Tonkovic S, Ostojic S (2009). Surface EMG based muscle fatigue evaluation in biomechanics. Clin. Biomech. (Bristol, Avon).

[39] Phinyomark A, Thongpanja S, Hu H, Phukpattaranont P, Limsakul C (2012). The usefulness of mean and median frequencies in electromyography analysis. INTECH.

[40] Tamaki T, Uchiyama S (1995). Absolute and relative growth of rat skeletal muscle. Physiol. Behav..

[41] Changsiripun C, Yabushita T, Soma K (2009). Masticatory function and maturation of the jaw-opening reflex. Angle Orthod..

[42] Seki Y, Ishii N, Toda K, Soma K (2002). Influence of occlusal hypofunction induced by opposed tooth loss on periodontal mechanoreceptors in rat molars. Jpn. I. Oral Biol..

[43] Liu ZJ, Ikeda K, Harada S, Kasahara Y, Ito G (1998). Functional properties of jaw and tongue muscles in rats fed a liquid diet after being weaned. J. Dent. Res..

[44] Kitagawa Y (2004). Alterations in enzyme histochemical characteristics of the masseter muscle caused by long-term soft diet in growing rabbits. Oral Dis..

[45] Trulsson M (2006). Sensory-motor function of human periodontal mechanoreceptors. J. Oral Rehabil..

[46] Miyamoto K (1999). Masseter muscle activity during the whole day in children and young adults. J. Oral Rehabil..

[47] Grunheid T, Brugman P, Zentner A, Langenbach GE (2010). Changes in rabbit jaw-muscle activity parameters in response to reduced masticatory load. J. Exp. Biol..

[48] Seven YB, Mantilla CB, Zhan WZ, Sieck GC (2013). Non-stationarity and power spectral shifts in EMG activity reflect motor unit recruitment in rat diaphragm muscle. Respir. Physiol. Neurobiol..

[49] Tsai CY, Lin YC, Su B, Yang LY, Chiu WC (2012). Masseter muscle fibre changes following reduction of masticatory function. Int. J. Oral Maxillofac. Surg..

[50] Komino M, Shiga H (2017). Changes in mandibular movement during chewing of different hardness foods. Odontology.

[51] Grunheid T, Langenbach GE, Korfage JA, Zentner A, van Eijden TM (2009). The adaptive response of jaw muscles to varying functional demands. Eur. J. Orthod..

[52] Peyron MA, Lassauzay C, Woda A (2002). Effects of increased hardness on jaw movement and muscle activity during chewing of visco-elastic model foods. Exp. Brain Res..

[53] Sato I, Konishi K (2004). Effects of soft diet on rat masseter muscle mitochondrial development. Okajimas Folia Anat. Jpn..

[54] Miyata H, Sugiura T, Kawai Y, Shigenaga Y (1993). Effect of soft diet and aging on rat masseter muscle and its motoneuron. Anat. Rec..

[55] Kato T (2015). Anatomical organization of descending cortical projections orchestrating the patterns of cortically induced rhythmical jaw muscle activity in guinea pigs. Neurosci. Res..

[56] Morquette P (2012). Generation of the masticatory central pattern and its modulation by sensory feedback. Prog. Neurobiol..

[57] Brocard F, Verdier D, Arsenault I, Lund JP, Kolta A (2006). Emergence of intrinsic bursting in trigeminal sensory neurons parallels the acquisition of mastication in weanling rats. J. Neurophysiol..

[58] Adachi K, Lee JC, Hu JW, Yao D, Sessle BJ (2007). Motor cortex neuroplasticity associated with lingual nerve injury in rats. Somatosens. Mot. Res..

[59] Paxinos G, Watson C (2007). The Rat Brain in Stereotaxic Coordinates.

