# Supplementary material for: Effects of low occlusal loading on the neuromuscular behavioral development of cortically-elicited jaw movements in growing rats
Source: Sci Rep. 2021 Mar 30;11:7175. doi: 10.1038/s41598-021-86581-9 (PMC8010060; doi:10.1038/s41598-021-86581-9)
Supplement: Supplementary file 1 — Supplementary Information. [file 41598_2021_86581_MOESM1_ESM.docx]

**Effects of low occlusal loading on the neuromuscular behavioral development of cortically-elicited jaw movements in growing rats**

Phyo Thura Aung^a^, Chiho Kato^a^*, Akiyo Fujita^a^, Yasunori Abe^a^, Takuya Ogawa^a^,

Hideyuki Ishidori^a^, Hidemasa Okihara^a^, Satoshi Kokai^a^, Takashi Ono^a^

^a^Department of Orthodontic Science, Graduate School of Medical and Dental Sciences, Tokyo Medical and Dental University (TMDU), Tokyo, Japan

***Corresponding author**

Chiho Kato, D.D.S., Ph.D.

1-5-45 Yushima Bunkyo-ku, Tokyo 113-8549, Japan

TEL/FAX: +03-5803-5530

[katorts@tmd.ac.jp](mailto:katorts@tmd.ac.jp)

This file includes: Supplementary figure 1-3, Supplementary table 1.


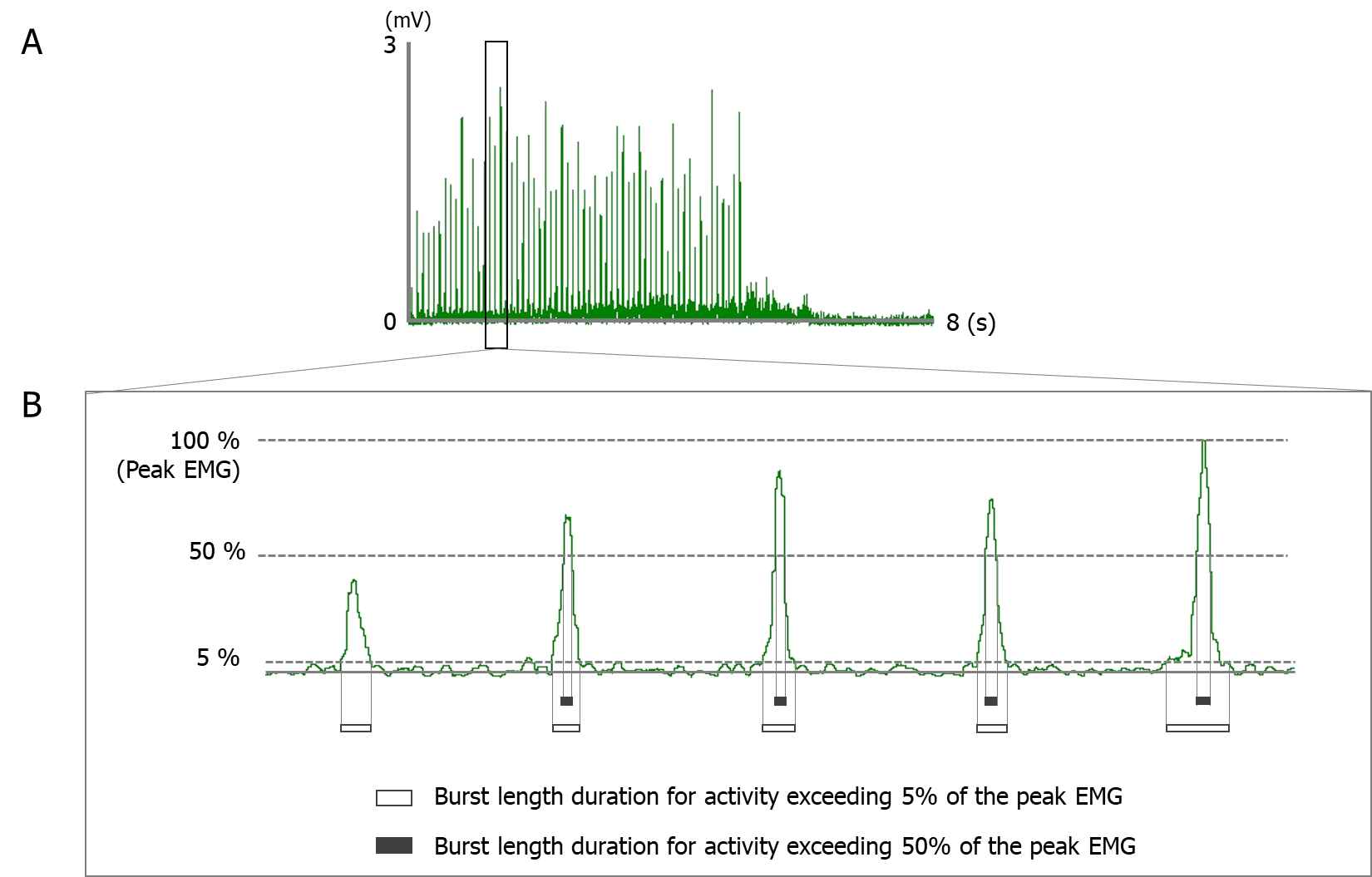


**Supplementary Figure 1**. **Typical example of rectified electromyographic (EMG) activity and details of the quantification of individual bursts.**

Rectified EMG recorded from the of anterior digastric muscle (A) and expanded view from the rectified EMG clarifies the method for determining the burst length at different levels of peak activity (B).


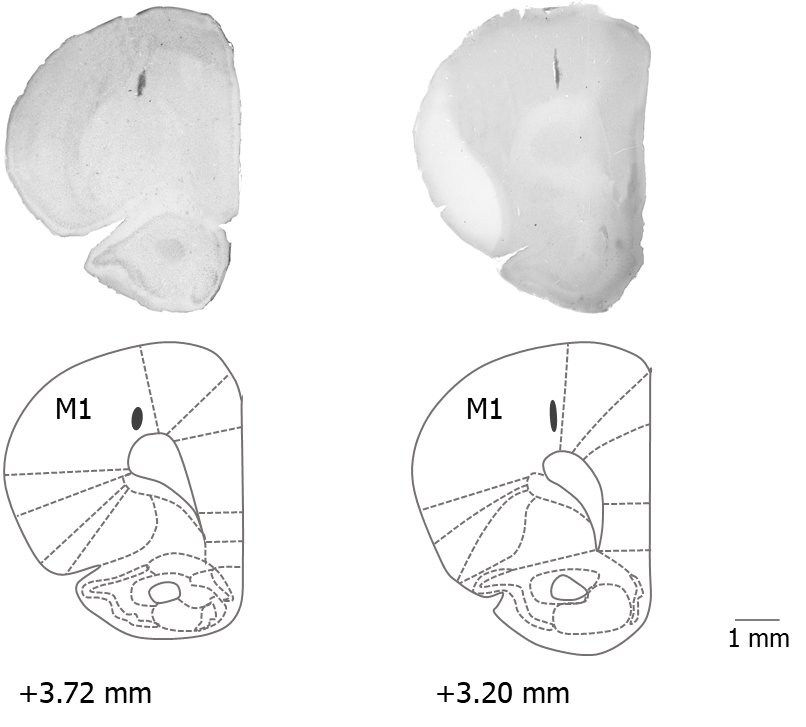


**Supplementary Figure 2**. **Coronal section of hematoxylin-eosin stain with schematic drawing of the stimulation sites in the anterior part of the left cortical masticatory area (A-CMA).**

Relative distances from Bregma in the rostral (+3.72 and +3.20 mm anterior to the Bregma) direction are depicted. Template from the brain atlas (Paxinos and Watson, 2007), illustrating the left primary motor cortex (anterior region of CMA) and showing the location of stimulation sites. 50 µm in thickness. Abbreviation: M1, primary motor cortex.


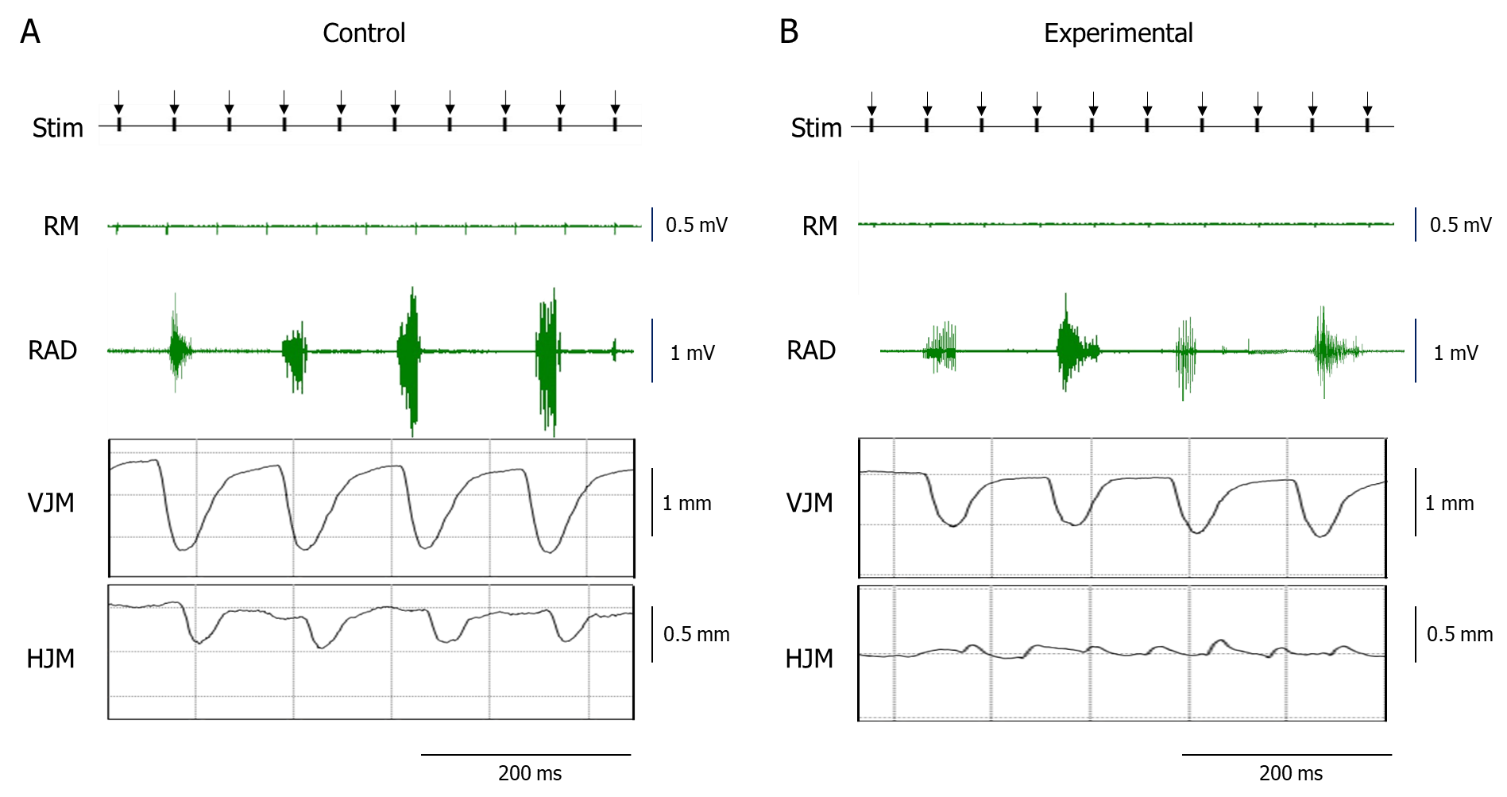


**Supplementary Figure 3. Typical example of enlarged raw data including electromyographic activity and jaw movement patterns.**

Control (A) and experimental (B) groups are shown. Downward arrows indicate the timing of electrical stimulation. Abbreviation: Stim, electrical stimulation; RM, right masseter muscle; RAD, right anterior digastric muscle; VJM, vertical jaw movements; HJM, horizontal jaw movements.

| Parameters | Definition |
| --- | --- |
| 1. Onset latency (ms) | Interval between the onset of the stimulus and the onset of the first response. |
| 1. Peak-to-peak amplitude (mV) | Amplitude from baseline to the positive peak of the response. |
| 1. Duty time (%) | Relative times at the 5%, 20%, and 50% activity level are the percentage duration of all EMG activity during stimulated contraction. |
| 1. Mean burst length (ms) | Total mean duration of muscle activities exceeding 5%, 20%, and 50% of peak EMG activity during stimulated contraction. |
| 1. Median frequency of EMG activity (Hz) | EMG power spectrum divided into two parts with equal amplitude. |
| 1. Mean frequency of EMG activity (Hz) | Average frequency from the sum of the product of the EMG. Power spectrum and the frequency divided by the total sum of the power spectrum. |
| Parameters used in electromyographic activity analysis | |
| 1. Gape size | Vertical excursion between maximum opening and maximum closing. |
| 1. Lateral excursion | Horizontal distance between minimum jaw-opening position and the most lateral jaw position. |
| 1. Changes in pattern of jaw movements | The 13 points traced with an interval of 10 ms along the path of one cycle of vertical jaw movement. |
| 1. Rhythm of jaw movements | Total cycle duration of the jaw movement during stimulation in each time point per second. |
| 1. Jaw-opening duration | Time between maximum closing and the subsequent maximum opening. |
| 1. Jaw-closing duration | Time between maximum opening and the subsequent maximum closing. |

**Supplementary Table 1**. **Parameters used to analyze jaw movement and electromyography activity**
